# Focused ultrasound in neuro-oncology: the role of the Focused Ultrasound Foundation in driving adoption and innovation

**DOI:** 10.1007/s11060-021-03808-5

**Published:** 2021-08-03

**Authors:** Emily C. Whipple, Camille A. Favero, Neal F. Kassell

**Affiliations:** Focused Ultrasound Foundation, Charlottesville, VA USA

## Abstract

The Focused Ultrasound Foundation was created to improve the lives of millions of people worldwide by accelerating the development of this noninvasive technology. The Foundation works to clear the path to global adoption by organizing and funding research, fostering collaboration, and building awareness among patients and professionals. Since its establishment in 2006, the Foundation has become the largest nongovernmental source of funding for focused ultrasound research. For more information, visit http://www.fusfoundation.org.

The evolution of any new, highly disruptive, therapeutic technology like focused ultrasound—from laboratory research to widespread utilization as a mainstream standard of care—is a glacial process that often takes decades. Knowing this, when we started the Focused Ultrasound Foundation 15 years ago, we first analyzed the ecosystem to identify all of the relevant organizations and the requirements of each to move this technology from the bench to the bedside. In doing so we learned that this extraordinarily complicated process involves a large number of very difficult steps, to be accomplished by a bewildering array of organizations within the ecosystem—most of whom also have different agendas, different timelines, and different decision-making processes. Additionally, multiple fairly daunting impediments exist which further compound the lab to clinic process including: lack of awareness of the potential of the technology; the need for long term, rigorous scientific evidence of safety, efficacy, access, and cost; regulatory approvals, which take a long time to achieve; insurance reimbursement, which can take even longer; and the fact that physicians are notoriously resistant to change, and often part of fairly vicious turf battles between the various medical specialties.

We created the Foundation to help the field navigate these obstacles and ultimately achieve our mission of **accelerating the development and adoption of focused ultrasound worldwide**. Created in October 2006, the Foundation is a tax exempt 501(c)3 organization facilitating medical research, education, and advocacy. It operates as a high performance, high impact, high technology service organization, and—much like a venture-backed organization in the private sector—the Foundation is market driven and action and results oriented. As such, the culture, systems, and processes of the Foundation would be found in a similar-scale service organization functioning at the highest level in the private sector. With our primary offices located in Charlottesville, VA, and subsidiary foundations in London and Hong Kong, the Foundation has a strong global influence and is being increasingly recognized as a model of a social entrepreneurship organization: it was recognized in 2018 by Charity Navigator as one of the top 10 medical research organizations in America, and is taught as an exemplar at the Batten School of Leadership and Public Policy and the Darden School of Business at the University of Virginia.

The way the Foundation operates is unique because the field is changing so rapidly. On a frequent and ongoing basis, we conduct analyses of the critical path from laboratory research to widespread utilization to identify current roadblocks; we then apply resources to overcome those barriers and take advantage of opportunities. We also engage in a wide variety of strategic activities to help accomplish our mission: as the guiding light or compass for the entire field, the Foundation helps identify critical unmet clinical needs and set research priorities for the technology’s various mechanisms of action and clinical indications; we work hard to change the culture in the ecosystem, to make all of the stakeholders more patient-centric with a sense of urgency and a sense of collaboration; we orchestrate the flow of information; and we spend a lot of time, money, and energy breaking down the silos of secrecy between various government agencies, academic institutions, manufacturers, and associated industrial organizations in order to foster collaboration, stimulate innovation, and rapidly achieve a critical mass of knowledge—all to accelerate the development and adoption of focused ultrasound as a standard of care.

The cornerstone of all of the Foundation’s activities is our research program, which since inception has included nearly 200 research projects at 55 institutions in 13 countries around the world. As the largest non-government source of funding for focused ultrasound research—totaling nearly $25 million and counting—the Foundation organizes, conducts, and funds research to compile the evidence of feasibility, safety, efficacy, access, and cost that is needed by all the stakeholders. We also offer a fellowship program that brings to Charlottesville mid-career clinicians and scientists from around the world for one to 2 years, and then they return to their home institution having established a sound basis for long term collaboration. The Foundation hosts two robust internship programs as well—one in Charlottesville, which runs year-round, and another global program for the summer, in which approximately 20 of the leading focused ultrasound investigators partner with one or two students in their institution for the summer months—all under the rubric of cultivating the next generation. New in 2021 is the Andrew J. Lockhart Postdoctoral Fellowship in Focused Ultrasound and Immuno-Oncology, a 1-year fellowship that pairs focused ultrasound investigators with immunology laboratories, and/or immuno-oncology investigators with focused ultrasound laboratories, to continue identifying and cultivating the best minds in the field, both in the US and abroad.

In terms of orchestrating the flow of information within the ecosystem, the Foundation aggregates and shares knowledge through its robust website, which attracts more than 27,000 visitors per month and is widely recognized as the “encyclopedia of focused ultrasound” with the most comprehensive, trusted, and up-to-date information. Webinars are also hosted approximately once per month on topics related to focused ultrasound such as cancer immunotherapy and blood–brain barrier opening. Our e-newsletter is distributed biweekly to thousands around the globe, and we raise awareness of focused ultrasound through diverse and targeted media outreach to print, broadcast, and digital outlets, and via social media.

Critical to fostering collaboration and sharing information is our International Symposium on Focused Ultrasound, the field’s largest and most important meeting, which has been organized and sponsored by the Foundation every 2 years since 2008. Historically it has been in the Washington, DC, area; this past year our 7th annual symposium pivoted to a virtual meeting due to the COVID-19 pandemic and welcomed more than 1800 participants from 58 countries. We also organize several smaller invitation-only workshops every year, bringing together leading figures from industry, academia, The National Institutes of Health, the US Food and Drug Administration, clinicians, scientists, physicists, and others to develop a roadmap of technology, development, preclinical laboratory studies, and clinical trials to lead to new clinical indications for focused ultrasound in the shortest possible time. The Foundation also sponsors meetings for associated organizations to continue to increase awareness of the technology, interface with clinicians and scientists, and support programs on discussion of the technology.

The Foundation’s Center of Excellence Program, established in 2009, brings together the best people and technical resources at select luminary sites in a multi-disciplinary environment. The Centers are created through partnerships of academia, industry, and the Foundation to showcase the technology and serve as hubs for collaboration. There are currently 11 (and counting) Centers of Excellence around the world, including the two newest in the Netherlands and Washington, DC.

As technological advances and awareness must support a robust and healthy industrial fabric, the Foundation has partnered with the approximately 55 focused ultrasound manufacturers through our FUS Partners program. FUS Partners provides integral support to manufacturers by sharing the Foundation’s domain expertise and institutional knowledge through a variety of activities—from regulatory and reimbursement assistance to strategic partnerships and advocacy. Finally, the Foundation also facilitates commercialization of the technology by working with the FDA to help them become a bridge, rather than a barrier, to the adoption of the technology.

To accomplish its mission, the Foundation has created an extraordinary team of athletes who can play more than one position. The organization and the team have been built with the philosophy that As attract As, and Bs attract Cs. Additionally, to guide the Foundation there is an exceptional board of directors of actively engaged and passionate individuals that represent virtually every discipline that the Foundation needs. It is not a typical philanthropic board—nobody is obliged to donate and there is no social cache—and it is truly a corporate governance board that represents the interests of the stakeholders. The Foundation also works closely with its Council which, in essence, is its “goodwill SWAT team,” comprised of enthusiastic individuals who make introductions to people of influence, help spread the word about focused ultrasound, support the Foundation, and on a frequent basis, host awareness and/or fundraising events.

In 2021, we have recently passed the inflection point on the adoption curve for focused ultrasound, where the dialogue has shifted from “if” the technology is going to have an important role in the therapeutic armamentarium, to “when”—from if to when. But more importantly, we are just at the beginning of the transition point from primarily a research environment to the beginning of a *patient treatment* environment—from science to sales. To bring focused ultrasound across the finish line, we have to do five things: One is to provide **evidence** of feasibility, safety, efficacy, access, and cost for all the stakeholders; the second is to build **awareness**; third is **capital**, both financial capital to support research and fund the manufacturers, as well as human or intellectual capital; the fourth is promoting successful **manufacturers**—the Foundation’s vision of improving the lives of millions of people with a wide variety of serious medical disorders *cannot be achieved* without successful commercial organizations to manufacture and distribute the equipment; and lastly, because no single entity can drive the acceleration of focused ultrasound, we need **partnerships** to successfully drive forward innovation and rapidly scale the technology.

It is widely understood that focused ultrasound has the potential to transform the treatment of a variety of serious medical conditions, including cancer (such as glioblastoma), Alzheimer’s disease, depression, epilepsy, and many more. All indicators point toward the evolution of this platform technology into a robust medical field, with the pace of research and development, publications, patient treatments, and the number of device manufacturers all increasing rapidly in the past few years. For example, when the Foundation first started there were just three mechanisms of action for the technology; today there are more than 30. There are also currently more than 130 different clinical indications for focused ultrasound in various stages of development; sufficient evidence has led to regulatory approvals around the world for more than 30 indications, seven of which are in the United States. Reimbursement has also been achieved for many of the approved indications, but not all. And focused ultrasound commercial treatments are now available in approximately 800 facilities around the world—with 180 in North America.

The vision is that by 2035 more than one million patients will be treated with focused ultrasound each year in at least 10,000 facilities worldwide. The goal of the Foundation and the field is clear. Our tasks are many. By banding together, working hard, and taking risks—while collectively directing our efforts on evidence, awareness, capital, businesses, and partners—the focused ultrasound community can and will cross the finish line, literally changing the future of healthcare and improving the lives of millions of people around the world.

