# Correction to: Focused ultrasound in neuro‑oncology: the role of the Focused Ultrasound Foundation in driving adoption and innovation

**DOI:** 10.1007/s11060-021-03821-8

**Published:** 2021-08-16

**Authors:** Emily C. Whipple, Camille A. Favero, Neal F. Kassell

**Affiliations:** Focused Ultrasound Foundation, Charlottesville, VA USA

## Correction to: Journal of Neuro-Oncology 10.1007/s11060-021-03808-5

Due to a processing error, this article was initially published including the wrong Abstract. The correct Abstract is shown below, and the original article has been corrected.

## Abstract

The Focused Ultrasound Foundation was created to improve the lives of millions of people worldwide by accelerating the development of this noninvasive technology. The Foundation works to clear the path to global adoption by organizing and funding research, fostering collaboration, and building awareness among patients and professionals. Since its establishment in 2006, the Foundation has become the largest nongovernmental source of funding for focused ultrasound research. For more information, visit http://www.fusfoundation.org.

